# Apology

**Published:** 1855-07

**Authors:** 


### APOLOGY.

We tender an apology to our esteemed cotemporary, the New Jersey Medical Reporter, for omitting to acknowledge in our last issue our indebtedness to the proof-sheets he so kindly sent us, for the full and excellent report we furnished our readers, of the Transactions of the American Medical Association. When the Association meets in Louisville we hope to have it in our power to reciprocate the kindness. D. W. Y.

